# Intermolecular contributions, filtration effects and signal composition of SIFTER (single-frequency technique for refocusing)

**DOI:** 10.5194/mr-4-1-2023

**Published:** 2023-01-12

**Authors:** Agathe Vanas, Janne Soetbeer, Frauke Diana Breitgoff, Henrik Hintz, Muhammad Sajid, Yevhen Polyhach, Adelheid Godt, Gunnar Jeschke, Maxim Yulikov, Daniel Klose

**Affiliations:** 1 Laboratory of Physical Chemistry, ETH Zürich, Vladimir-Prelog-Weg 2, 8093 Zurich, Switzerland; 2 Department of Chemistry, Bielefeld University, Universitätsstrasse 25, 33615 Bielefeld, Germany

## Abstract

To characterize structure and molecular order in the nanometre range, distances between electron spins and their distributions can be measured via dipolar spin–spin interactions by different pulsed electron paramagnetic resonance experiments. Here, for the single-frequency technique for refocusing dipolar couplings (SIFTER), the buildup of dipolar modulation signal and intermolecular contributions is analysed for a uniform random distribution of monoradicals and biradicals in frozen glassy solvent by using the product operator formalism for electron spin S=1/2 . A dipolar oscillation artefact appearing at both ends of the SIFTER time trace is predicted, which originates from the weak coherence transfer between biradicals. The relative intensity of this artefact is predicted to be temperature independent but to increase with the spin concentration in the sample. Different compositions of the intermolecular background are predicted in the case of biradicals and in the case of monoradicals. Our theoretical account suggests that the appropriate procedure of extracting the intramolecular dipolar contribution (form factor) requires fitting and subtracting the unmodulated part, followed by division by an intermolecular background function that is different in shape. This scheme differs from the previously used heuristic background division approach. We compare our theoretical derivations to experimental SIFTER traces for nitroxide and trityl monoradicals and biradicals. Our analysis demonstrates a good qualitative match with the proposed theoretical description. The resulting perspectives for a quantitative analysis of SIFTER data are discussed.

## 1 Introduction

Distances between electron spins (and in particular distance distributions) are an important source of information for different research fields, ranging from structural biology of ordered and disordered proteins [Bibr bib1.bibx41] to supramolecular chemistry and material science [Bibr bib1.bibx38]. Distance distributions in the nanometre range are accessible by pulsed dipolar spectroscopy (PDS), which is an increasingly applied group of techniques in the field of pulsed EPR spectroscopy [Bibr bib1.bibx23]. PDS offers a number of strategies for inter-spin distance determination, of which to date the most frequently applied PDS experiment is four-pulse DEER [Bibr bib1.bibx33]. In the double-resonance experiment DEER, the spectrum is separated into two fractions of spin packets excited at different frequencies [Bibr bib1.bibx22]. Contrary to this, single-frequency experiments [Bibr bib1.bibx5] strive to excite the whole spectrum of coupled spin pairs and depend on coherence transfers of both coupled spins that are excited by the same pulses. The most well-known examples of this class of experiments are the six-pulse DQC [Bibr bib1.bibx4] and the four-pulse SIFTER [Bibr bib1.bibx24] sequences, with the latter being discussed here.

With the advent of ultra-wideband EPR spectrometers as well as novel spin labels, in particular based on the trityl radical with its narrow EPR spectrum, single-frequency PDS techniques find broader applications [Bibr bib1.bibx29]. In theory, single-frequency experiments are advantageous, particularly when applied to narrow lines, as they do not suffer from problems such as limited modulation depth due to separation into spin packets or pulse overlap leading to artefacts as known for DEER. While significant efforts need to be made in order to garner the advantages of the SIFTER experiment for nitroxide spin labels, single-frequency experiments are the preferred choice for spin systems with more narrow line widths. One such class of systems is trityl radicals – carbon-centred organic radicals based on which numerous spin labels have been developed [Bibr bib1.bibx26], as their high reduction stability and long decoherence times make them potentially suitable for room-temperature as well as in-cell distance measurements [Bibr bib1.bibx37]. In applications using trityls it has been shown that SIFTER is significantly superior to double-frequency experiments [Bibr bib1.bibx31], unless when measuring at very high magnetic fields, where the line width is sufficiently increased due to its dependence on g anisotropy and where available microwave power and bandwidth may be insufficient to enable efficient excitation in single-frequency experiments [Bibr bib1.bibx2].

However, while contributions to the DEER signal including artefacts have been described theoretically [Bibr bib1.bibx32] and can be fitted almost perfectly [Bibr bib1.bibx13], single-frequency pulsed dipolar spectroscopy techniques exhibit a signal decay behaviour that cannot be described through models of coupled spins in a random spin bath as used, for example, for the analytical description of backgrounds in DEER sequences. A clear mathematical separation into intramolecular dipolar signal (a.k.a. form factor) and intermolecular contribution to the dipolar signal (a.k.a. background) has so far not been accomplished. From the application perspective, the unknown intermolecular background in the dipolar evolution data from SIFTER presents a severe limitation as it hampers reliable distance determination from SIFTER time-domain data [Bibr bib1.bibx24]. Experimental efforts have been made to reduce the background by exciting a larger fraction of the spectral line through application of frequency-swept pulses in the experiment [Bibr bib1.bibx11]. In doing so, it has been shown that information on orientation selection can also be made accessible through SIFTER [Bibr bib1.bibx11]. The main approach so far for the SIFTER intermolecular background correction has been based on two heuristic assumptions [Bibr bib1.bibx46]. First, that the background function can be factorized analogously as in DEER; second, it has been recognized that the background must contain an additional term, for which similarity in shape has been found to the SIFTER-delay refocused echo (SIDRE) sequence trace (see Fig. [Fig Ch1.F1]a) [Bibr bib1.bibx46]. Alternatively, the background is fitted with Gaussian or stretched exponential models, with the latter having been shown to be more suitable [Bibr bib1.bibx7]. Yet, in spite of best efforts, background uncertainty still remains.

In this work, we derive a theoretical model for the intermolecular background of the SIFTER experiment based on dipolar terms and product operator formalism for the evolution of the spin density operator. After the mathematical derivation, we go on to compare this model to experimental data on nitroxides and trityls, both as monoradicals and biradicals.

## 2 Derivation

The derivation section consists of three main parts. First, we briefly summarize the formation of the SIFTER signal in a sample consisting of isolated pairs of spins (intramolecular contributions only), according to the original derivation [Bibr bib1.bibx24]. On this occasion we also discuss the topology of spin operator terms in the spin density matrix throughout the sample. Second, we discuss the intermolecular dipolar evolution in the SIFTER experiment on a frozen solution of monoradicals. We also discuss filtering effects due to the electron–nuclear interactions and distribution of transverse evolution times, as well as the structure of the intermolecular SIFTER signal appearing due to these filtering effects. Third, for a frozen solution of biradicals, we follow the propagation of the density operator in the SIFTER pulse sequence when both intra- and intermolecular spin couplings are present. In this part, we first follow the terms of the density matrix that eventually produce the correct dipolar modulation, with the properties analogous to those of the DEER experiment. Other relevant terms leading to detectable signals are mentioned but kept aside. Next, we consider these additional terms appearing due to the intermolecular coherence transfer, show that they should produce an artefact at the two ends of the SIFTER time trace, and qualitatively discuss the temperature and concentration dependence of this artefact. Finally, similar to the case of monoradical solutions, we discuss for biradicals the filtering effects and additional artefacts appearing in this case.

**Figure 1 Ch1.F1:**
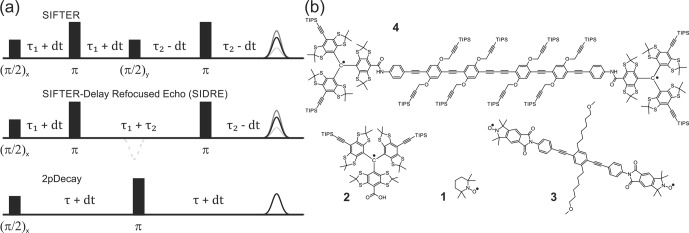
Pulse sequences used in experiments are shown in **(a)**. The time axis in SIFTER traces is $t=\tau_{1}-\tau_{2}$ , and the interpulse delays are incremented/decremented by d t to keep the total transverse evolution time $2\tau_{0}=2(\tau_{1}+\tau_{2})$ constant. The chemical structures of the studied compounds are depicted in **(b)**. Nitroxide monoradical (TEMPO) (1), trityl monoradical (2), nitroxide biradical (3) and trityl biradical (4). TIPS is triisopropylsilyl.

We restrict our derivation to the four-pulse SIFTER sequence shown in Fig. [Fig Ch1.F1]. The time axis in SIFTER traces is $t=\tau_{1}-\tau_{2}$ , and the interpulse delays are varied to keep the total transverse evolution time $2\tau_{0}=2(\tau_{1}+\tau_{2})$ constant. Throughout our calculations the following approximations are assumed to hold true:

i. All pulses are ideal (infinitely short with infinite excitation bandwidth).
ii. We analyse SIFTER experiments on frozen solutions of biradicals prepared such that the intramolecular dipole–dipole interaction is much stronger than the intermolecular dipole–dipole interactions (high dilution).
iii. We take into account only the secular part of the dipolar couplings, which for a pair of spins A and B is written as
1 $${\hat{H}}_{\mathrm{dd},\sec}=\omega_{\mathrm{dd}}(r,\theta )\cdot {\hat{S}}_{A,z}{\hat{S}}_{B,z}\;.$$
  Here, $\omega_{\mathrm{dd}}(r,\theta )$ is the secular element of the electron–electron magnetic dipolar coupling in frequency units, which is distance and orientation dependent. This is equivalent to the assumption that the dipolar frequencies are much smaller than the width of the corresponding EPR spectrum, as in this case, spin pairs are rare for which the flip-flop term of the dipole–dipole coupling needs to be considered.

Also, we assume for simplicity that all spin centres in the sample have the same EPR spectrum. This is, however, never explicitly used in the derivation, which therefore holds true also for SIFTER with heterogeneous spin pairs, should such an experiment appear to be of interest.

### 2.1 SIFTER in an ensemble of isolated spin pairs (intramolecular contribution)

In the calculations here and in the sections below we can drop the electron Zeeman interaction, since it is refocused at the echo positions. Thus, in the case of an isolated spin pair, the spin Hamiltonian is simply given by Eq. ([Disp-formula Ch1.E1]).

The four-pulse SIFTER pulse sequence contains two τ -( π )- τ refocusing blocks, where ( π ) stands for a π pulse, and τ stands for a delay of duration τ . In between these two blocks a phase-shifted π/2 pulse is inserted, which is responsible for the coherence transfer between dipolar coupled spin pairs that leads to a solid echo. The overall propagation of the spin density matrix for a statistically large ensemble of non-interacting biradicals can be described as follows [Bibr bib1.bibx24].

Initially, before the primary $(\pi /2{)}_{x}$ pulse, the magnetization is aligned with the static magnetic field, and the density operator can be written as

2 $${\hat{\sigma }}_{s}(-\delta t)=-{\hat{S}}_{A,z}-{\hat{S}}_{B,z}.$$

The time δt stands for an infinitely short period of evolution time. Indices A and B correspond to the two spins in a given biradical. These can be chemically identical moieties, distinguished only in theory by their spatial positions and orientation, which are fixed in a frozen glassy sample. Obviously, in such a case the assignment of a particular spin to be A or B spin is arbitrary. We only state that we keep this assignment unchanged throughout the calculation. Just after the primary $(\pi /2{)}_{x}$ pulse, the magnetization is along the +y direction:

3 $${\hat{\sigma }}_{s}(\delta t)={\hat{S}}_{A,y}+{\hat{S}}_{B,y}.$$

The first block $\tau_{1}$ - (π) - $\tau_{1}$ refocuses the evolution under the electron Zeeman interactions for the two spins, but it keeps the evolution under the dipolar Hamiltonian. Just before the third pulse, the spin density operator is

4 $$\begin{array}{rl} {\hat{\sigma }}_{s}(2\tau_{1}-\delta t) & =-\mathrm{cos}(\omega_{\mathrm{dd}}\tau_{1})({\hat{S}}_{A,y}+{\hat{S}}_{B,y})\\ & +\mathrm{sin}(\omega_{\mathrm{dd}}\tau_{1})(2{\hat{S}}_{A,x}{\hat{S}}_{B,z}+2{\hat{S}}_{A,z}{\hat{S}}_{B,x}). \end{array}$$

The term ${\hat{S}}_{A,y}$ in the first parentheses and the term $2{\hat{S}}_{A,x}{\hat{S}}_{B,z}$ in the second parentheses result from the evolution of the term ${\hat{S}}_{A,y}$ in Eq. ([Disp-formula Ch1.E3]). Accordingly, the term ${\hat{S}}_{B,y}$ in the first parentheses and the term $2{\hat{S}}_{A,z}{\hat{S}}_{B,x}$ in the second parentheses result from the evolution of the term ${\hat{S}}_{B,y}$ in Eq. ([Disp-formula Ch1.E3]). The $(\pi /2{)}_{y}$ pulse inverts the signs and swaps the two terms in the second parentheses; thus, the term $2{\hat{S}}_{A,x}{\hat{S}}_{B,z}$ is transformed into $-2{\hat{S}}_{A,z}{\hat{S}}_{B,x}$ , and the term $2{\hat{S}}_{A,z}{\hat{S}}_{B,x}$ is transformed into $-2{\hat{S}}_{A,x}{\hat{S}}_{B,z}$ :

5 $$\begin{array}{rl} {\hat{\sigma }}_{s}(2\tau_{1}+\delta t) & =-\mathrm{cos}(\omega_{\mathrm{dd}}\tau_{1})({\hat{S}}_{A,y}+{\hat{S}}_{B,y})\\ & -\mathrm{sin}(\omega_{\mathrm{dd}}\tau_{1})(2{\hat{S}}_{A,z}{\hat{S}}_{B,x}+2{\hat{S}}_{A,x}{\hat{S}}_{B,z})\;. \end{array}$$

Finally, the second evolution block $\tau_{2}$ - (π) - $\tau_{2}$ leads to the SIFTER signal in the form

6 $$\begin{array}{rl} {\hat{\sigma }}_{s}(2\tau_{1}+2\tau_{2}) & =\mathrm{cos}(\omega_{\mathrm{dd}}(\tau_{2}-\tau_{1}))({\hat{S}}_{A,y}+{\hat{S}}_{B,y})\\ & -\mathrm{sin}(\omega_{\mathrm{dd}}(\tau_{2}-\tau_{1}))(2{\hat{S}}_{A,z}{\hat{S}}_{B,x}+2{\hat{S}}_{A,x}{\hat{S}}_{B,z})\;. \end{array}$$

Note for later discussions that the term $\mathrm{cos}(\omega_{\mathrm{dd}}(\tau_{2}-\tau_{1}))({\hat{S}}_{A,y}+{\hat{S}}_{B,y})$ comes from a sum of two parts:

7 $$\mathrm{cos}(\omega_{\mathrm{dd}}\tau_{1})\mathrm{cos}(\omega_{\mathrm{dd}}\tau_{2})({\hat{S}}_{A,y}+{\hat{S}}_{B,y})$$

and

8 $$\mathrm{sin}(\omega_{\mathrm{dd}}\tau_{1})\mathrm{sin}(\omega_{\mathrm{dd}}\tau_{2})({\hat{S}}_{A,y}+{\hat{S}}_{B,y})\;.$$

The cosine term results from the evolution that takes place always on the same spin, while the sine term results from evolution on the first spin during the first refocusing block, coherence transfer and evolution on the second spin during the second refocusing block.

Before we make a detailed calculation in the following sections, let us discuss the overall topology of the density matrix propagation solution for the SIFTER experiment in the case of many weakly interacting biradicals (real frozen solution case). First, we note that the solution in Eq. ([Disp-formula Ch1.E6]) consists of two contributions, which originate from the initial polarizations $-{\hat{S}}_{A,z}$ and $-{\hat{S}}_{B,z}$ on the spins A and B. Each of these two spin terms propagates independently from the other one; thus, the final equation can be obtained by propagating only one of these contributions and then adding the other one, which has an analogous structure of the spin operators, differing only by the change of indices. Second, in the case of interacting biradicals, more than one dipolar coupling term will affect the evolution of the density operator. All these terms, as long as we assume the secular approximation, will mutually commute. The evolution will lead to the coherence transfer not only within the A–B spin pair of the same molecule but also between spins that belong to different biradical molecules. These branched contributions with partial coherence on many spins will nevertheless stay additive with respect to the electron spins from which the magnetization originated, so the result of the propagation for each initial single-spin polarization can be computed independently and then added to other parts of the solution. This allows us to perform SIFTER sequence propagation for one arbitrarily chosen electron spin and then perform ensemble averaging of this solution. For the averaging, since we deal with biradicals, we must keep in mind that for each A-spin solution in the given biradical that we compute, the ensemble solution will contain the corresponding symmetric B-spin solution, which would then recover the symmetric form of the intramolecular SIFTER signal, analogous to the one given in Eq. ([Disp-formula Ch1.E6]).

### 2.2 The intermolecular part of the SIFTER signal

#### 2.2.1 SIFTER in a frozen monoradical solution

Before we discuss the case of SIFTER experiment in the presence of intramolecular spin–spin distances, we need to discuss an important case of SIFTER experiment in a frozen solution of monoradicals. A monoradical-like signal also appears in the SIFTER experiment on biradicals because of incomplete excitation of paramagnetic species [Bibr bib1.bibx24]. In other words, the SIFTER signal of a frozen solution of biradicals consists of a modulated part, which is the actual biradical signal, and a non-modulated part, which has the same properties as a frozen solution of monoradicals and, as we will show below, consists only of a sum of two different intermolecular contributions. In turn, we will demonstrate that the biradical signal is a sum of three intramolecular contributions, each multiplied with a somewhat different intermolecular decay function. We shall see in the following that the intermolecular contributions of the biradical signal (and of the monoradical signal) are not identical.

To introduce abbreviations consistent between monoradical and biradical cases, we consider one spin centre, called A spin, which has an initial polarization of $-{\hat{S}}_{A,z}$ . This spin operator is propagated in the SIFTER pulse sequence, and parts of the created coherence are transferred to other spins, called B spins. The B spin in the same biradical molecule will be marked with the index (0) , while all spins in the surrounding biradical molecules are assigned to be B spins with indices (i) , with i ranging from 1 to the total number of “intermolecular B spins” in the sample N . We further assume strong dilution, so intramolecular spin–spin coupling (in the case of biradicals) is much stronger than the intermolecular couplings. Let us adjust the notation for the spin operators and use the abbreviations ${\hat{S}}_{k}$ ( k=x,y,z ) for the A-spin operators, the abbreviations ${\hat{I}}_{k}^{\left(0\right)}$ for the partner B spin within the same biradical and the abbreviations ${\hat{I}}_{k}^{\left(l\right)}$ ( l=1…N ) for the remote B spins. Let us abbreviate dipolar frequency for the given conformation of biradical to be $\omega_{0}$ , while the corresponding intermolecular A–B dipolar frequencies are designated as $\omega_{l}$ ( N different frequencies), with the same meaning of index l as above for the spin operators. Furthermore, let us use the abbreviation ${\tilde{\omega }}_{l}$ for the intramolecular dipolar frequency in the molecule where the B spin with the index l is placed ( N/2 different frequencies), and let us use the abbreviations ${\tilde{\omega }}_{l,m}$ for the intermolecular dipolar frequency between spins l and m ( N(N+1)/2 different frequencies). Figure [Fig Ch1.F2] illustrates the different electron spin–spin coupling frequencies. Note that all dipolar Hamiltonian terms $2{\hat{S}}_{z}{\hat{I}}_{z}^{\left(l\right)}$ for l=0…N commute. Likewise, all operators describing the action of microwave pulses on different spins commute. In this section we drop the hyperfine interactions and consider a spin Hamiltonian for monoradicals that consists of only the electron spin–spin couplings:

9 $$\hat{H}=\sum_{l=1}^{N}\omega_{l}{\hat{S}}_{z}{\hat{I}}_{z}^{\left(l\right)}+\frac{1}{2}\sum_{i,j=1}^{N}{\tilde{\omega }}_{i,j}{\hat{I}}_{z}^{\left(i\right)}{\hat{I}}_{z}^{\left(j\right)}\;,$$

where the second sum runs over all pairs i≠j . As an overview, Table [Table Ch1.T1] summarizes the most important terms used or derived in the theory part of this paper.

**Figure 2 Ch1.F2:**
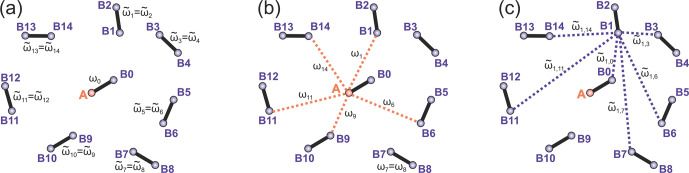
Intra- and intermolecular electron–electron coupling frequencies for the case of a frozen solution of biradicals. **(a)** Intramolecular dipolar frequency for the target biradical, containing the A spin ( $\omega_{0}$ ) and the intramolecular dipolar frequencies in other biradicals ( ${\tilde{\omega }}_{i}$ ), both spins in these biradicals are B spins. **(b)** Intermolecular dipolar frequencies $\omega_{i}$ describe couplings of A spin to remote B spins. **(c)** The dipolar frequencies ${\tilde{\omega }}_{i,j}$ describe intermolecular dipolar couplings between B spins. The case of a frozen solution of monoradicals is obtained by keeping only the intermolecular dipolar frequencies and dropping the intramolecular ones. Only a selection of intermolecular couplings is shown in the figures to reduce visual crowding.

**Table 1 Ch1.T1:** Most important terms describing the spin density matrix and the detected signal contributions.

Term	Equations	Description
Full density matrix or SIFTER signal and derivatives thereof		
${\hat{\sigma }}_{s}(t)$	2–6	Two-spin density matrix
${\hat{\sigma }}_{V}(t)$	10	Multi-spin density matrix
${\hat{\sigma }}_{V}^{\prime }(t)$	11–13	The part of the multi-spin density matrix leading to detectable terms
$V_{\mathrm{SIFTER}}(\tau_{1},\tau_{2})$	17, 32, 35	Full ensemble-averaged multi-spin SIFTER signal
$S_{\mathrm{SIFTER}}$	18, 36, 39	SIFTER signal $V_{\mathrm{SIFTER}}(\tau_{1},\tau_{2})$ divided by SIDRE signal $B_{S}(\tau_{1},\tau_{2})$
Parts of the detected SIFTER signal		
F(τ)	27…43	Intramolecular e – e dipolar term (Pake time-domain signal)
$B_{2p}(\tau )$	14, 15, 26, 27, 30, 34	Intermolecular e – e dipolar contribution (Hahn echo experiment)
$B_{t}(\tau )$	17, 18, 35, 36, 39–44	Intermolecular e – n contribution (Hahn echo experiment)
$B_{S}(\tau_{1},\tau_{2})$	17, 18, 35, 36, 39–44	Intramolecular e – n contribution (SIDRE experiment)

With this notation, in the case of a monoradical solution, evolution during the first $\tau_{1}$ - (π) - $\tau_{1}$ block results in the following terms:

10 $$\begin{array}{rl} {\hat{\sigma }}_{V}(2\tau_{1}-\delta t) & ={\hat{\sigma }}_{V,1}\\ & =-{\hat{S}}_{y}\prod_{l=1}^{N}\mathrm{cos}(\omega_{l}\tau_{1})\\ & +\sum_{n}\left[2{\hat{S}}_{x}{\hat{I}}_{z}^{\left(n\right)}\mathrm{sin}(\omega_{n}\tau_{1})\prod_{l\neq n}\mathrm{cos}(\omega_{l}\tau_{1})\right]\\ & +\hat{\Phi }\;. \end{array}$$

Here, all terms that contain products with more than one ${\hat{I}}_{z}$ operator are collected in the operator $\hat{\Phi }$ , which will not lead to detectable terms at the end of the SIFTER pulse sequence. Indeed, after the coherence transfer pulse, these terms will turn into products with two or more ${\hat{I}}_{x}$ operators (with different spin count indices l ), which cannot evolve into detectable single spin operators under the secular dipolar Hamiltonian. We will discuss this in more detail below on the example of biradicals. In the following we drop the term $\hat{\Phi }$ . Additionally, we substitute $\mathrm{sin}(\omega_{n}\tau_{1})$ by $\mathrm{tan}(\omega_{n}\tau_{1})\cdot \mathrm{cos}(\omega_{n}\tau_{1})$ . Like this, we can add the missing cosine term in the product. Also, assuming long intermolecular distances, we can then use Taylor series for the tan⁡ function, thus obtaining $\mathrm{tan}(\omega_{n}\tau_{1})\approx \omega_{n}\tau_{1}$ . This approximation is valid until $\omega_{n}\tau_{1}∼0.5$ , which would mean for a 10 µ s SIFTER trace bulk spin concentrations up to a few millimoles per litre, which is a good approximation under nearly any conditions used routinely in pulse EPR experiments on narrow-line radicals. Finally, the relevant part of the signal, which we will abbreviate as ${\hat{\sigma }}_{V,1}^{\prime }$ , is given by the following equation:

11 $$\begin{array}{rl} {\hat{\sigma }}_{V,1}^{\prime } & =-{\hat{S}}_{y}\prod_{l=1}^{N}\mathrm{cos}(\omega_{l}\tau_{1})\\ & +\sum_{n}\left[2{\hat{S}}_{x}{\hat{I}}_{z}^{\left(n\right)}\omega_{n}\tau_{1}\right]\prod_{l=1}^{N}\mathrm{cos}(\omega_{l}\tau_{1}). \end{array}$$

After the coherence transfer pulse, this transforms to

12 $$\begin{array}{rl} {\hat{\sigma }}_{V}^{\prime }(2\tau_{1}+\delta t) & ={\hat{\sigma }}_{V,2}^{\prime }\\ & =-{\hat{S}}_{y}\prod_{l=1}^{N}\mathrm{cos}(\omega_{l}\tau_{1})\\ & -\sum_{n}\left[2{\hat{S}}_{z}{\hat{I}}_{x}^{\left(n\right)}\omega_{n}\tau_{1}\right]\prod_{l=1}^{N}\mathrm{cos}(\omega_{l}\tau_{1}), \end{array}$$

and after the second evolution block $\tau_{2}$ - (π) - $\tau_{2}$ , this evolves to

13 $$\begin{array}{rl} {\hat{\sigma }}_{V}^{\prime }(2\tau_{1}+2\tau_{2}) & ={\hat{\sigma }}_{V,3}^{\prime }\\ & ={\hat{S}}_{y}\prod_{l=1}^{N}\mathrm{cos}(\omega_{l}\tau_{1})\prod_{l=1}^{N}\mathrm{cos}(\omega_{l}\tau_{2})\\ & +\sum_{n}\left[{\hat{I}}_{y}^{\left(n\right)}\omega_{n}^{2}\tau_{1}\tau_{2}\right]\prod_{l=1}^{N}\mathrm{cos}(\omega_{l}\tau_{1})\\ & \times \prod_{l=1}^{N}\mathrm{cos}({\tilde{\omega }}_{n,l}\tau_{2})+\hat{\Psi }. \end{array}$$

Here, the operator $\hat{\Psi }$ also accounts for all non-detectable spin operators. Note again that dipolar frequencies $\omega_{l}$ refer to the surrounding of the A spin, while the dipolar frequencies ${\tilde{\omega }}_{n,l}$ refer to the surrounding of the B spin with index n . In the list of surrounding spins for the B spin with the index n , we included the remaining N-1 B-spins and the A spin, so the total number of surrounding spins is again equal to N .

The product of cosine contributions from all surrounding spins, after ensemble averaging, describes the detectable signal in a Hahn echo experiment. We will use the abbreviation

14 $$B_{2p}(\tau )=\langle \prod_{l=1}^{N}\mathrm{cos}(\omega_{l}\tau )t\rangle .$$

Note that this product centred at the A spin should not correlate with the analogous product centred at the n th B spin. Therefore, we can assume that

15 $$\begin{array}{rl} & \langle \prod_{l}\mathrm{cos}(\omega_{l}\tau_{1})\prod_{m}\mathrm{cos}({\tilde{\omega }}_{n,m}\tau_{2})\rangle \\ & \;=\langle \prod_{l}\mathrm{cos}(\omega_{l}\tau_{1})\rangle \langle \prod_{m}\mathrm{cos}({\tilde{\omega }}_{n,m}\tau_{2})\rangle \\ & \;=B_{2p}(\tau_{1})B_{2p}(\tau_{2}). \end{array}$$

The function $B_{2p}(\tau )$ is the same as the well-known intermolecular background decay function in DEER, for the case of 100 % pump pulse inversion efficiency. This would be a mono-exponential decay function in the case of homogeneous spatial spin distribution, or it could be approximated as a stretched exponential function in the case of inhomogeneous distribution of spins [Bibr bib1.bibx35].

#### 2.2.2 Electron–proton contributions and transverse relaxation

Electron spins interact with surrounding nuclear spins and change their precession frequency according to the continuously ongoing configuration dynamics of the surrounding nuclei. It is common to call such a process spectral diffusion. By nature, this is a deterministic process, but due to the very large number of coupled nuclear spins and a random distribution of couplings, it demonstrates quasi-stochastic features. Accordingly, each pulse sequence can be seen as a path for a (quasi-)decay of electron coherence due to the nuclei-related dephasing, and at the same time it can be seen as a filter function, selecting certain electron–nuclear frequencies and suppressing others. The latter process is often called dynamical decoupling [Bibr bib1.bibx44]. The first interpretation can also be seen as a type of filtering, albeit a different one: local nuclear configurations around electron spins might differ in the characteristic decay times, due to the strengths of the couplings or due to the different statistics of nuclear spin-up and spin-down states. Thus, different pathways within one pulse sequence would have different suppression factors for electron spins in different nuclear surroundings. To make the formulations easier, we will simply refer to “filtering” of SIFTER signal contributions due to the electron–nuclear interactions without specifying the particular interpretation.

Here, we consider a spin Hamiltonian that besides electron–electron couplings also takes into account the electron–nuclear (hyperfine) interactions, nuclear Zeeman and nuclear spin–spin couplings:

16 $$\hat{H}=\sum_{l=1}^{N}\omega_{l}{\hat{S}}_{z}{\hat{I}}_{z}^{\left(l\right)}+\frac{1}{2}\sum_{i,j=1}^{N}{\tilde{\omega }}_{i,j}{\hat{I}}_{z}^{\left(i\right)}{\hat{I}}_{z}^{\left(j\right)}+{\hat{H}}_{e\text{-}n,n\text{-}n}.$$

Again, in the second sum we take all pairs i≠j . For brevity, we included all nuclei-related interactions into one Hamiltonian term ${\hat{H}}_{e\text{-}n,n\text{-}n}$ . The evolution due to the interaction with the nuclear bath cannot be computed analytically. Therefore, in the following, we will derive equations in analytical form by writing formally the corresponding decay functions obtained after the ensemble averaging of the detected signals. Here, we assume that while the electron–electron couplings are present (and they are responsible for the coherence transfer during the SIFTER experiment), these couplings make a relatively weak contribution to the overall decay, so the intermolecular decay is mainly due to the electron spin interactions with the nuclear bath. In the case when both electron–electron and electron–nuclear contributions make a comparable effect on the SIFTER decay, the results from this section need to be combined with the results from the previous section.

We can generally assume that during each echo refocusing block τ - π - τ , the intermolecular dipolar electron–electron contribution and other transverse relaxation contributions, such as the intrinsic $T_{2}$ relaxation and electron–proton contribution (spectral diffusion), are factorized. However, upon action of the central $(\pi /2{)}_{y}$ pulse, one part of the electron spin coherence is transferred to a different electron spin, while the other part remains on the same spin. In the case of a distribution of transverse relaxation properties in the ensemble of electron spins, some filtration effects would appear, and the mentioned two contributions of the SIFTER signal will have different shapes.

The first term in Eq. ([Disp-formula Ch1.E13]) corresponds to transverse evolution always on the same spin. Therefore, the second transverse evolution will happen already in a pre-filtered ensemble of electron spins, and its average transverse relaxation will thus be slower than for a common two-pulse echo decay. The overall dependence of this transverse decay contribution can be experimentally measured in a constant total time refocused echo experiment, which is equal to the SIFTER pulse sequence lacking the central coherence transfer pulse (the $(\pi /2{)}_{y}$ pulse). We shall abbreviate this transverse decay signal as $B_{S}(\tau_{1},\tau_{2})$ and refer to the corresponding experiment as SIDRE (SIFTER delay refocused echo). This is illustrated in Fig. [Fig Ch1.F3]a and c. The potential use of this experiment for SIFTER background correction has been mentioned before [Bibr bib1.bibx46]. The investigation of the refocused echo signal dependence on the two delay times indicates that the SIDRE signal has maximum intensity at $\tau_{1}=\tau_{2}$ , i.e. at $t=\tau_{1}-\tau_{2}=0$ , with t being the intrinsic SIFTER (and SIDRE) time variable [Bibr bib1.bibx3]. Note that, as indicated in the cited work, if either $\tau_{1}$ or $\tau_{2}$ is scanned without keeping the full evolution time $\tau_{0}=\tau_{1}+\tau_{2}$ constant, then the maximum spin echo intensity is observed at a time point different from $\tau_{1}=\tau_{2}$ .

**Figure 3 Ch1.F3:**
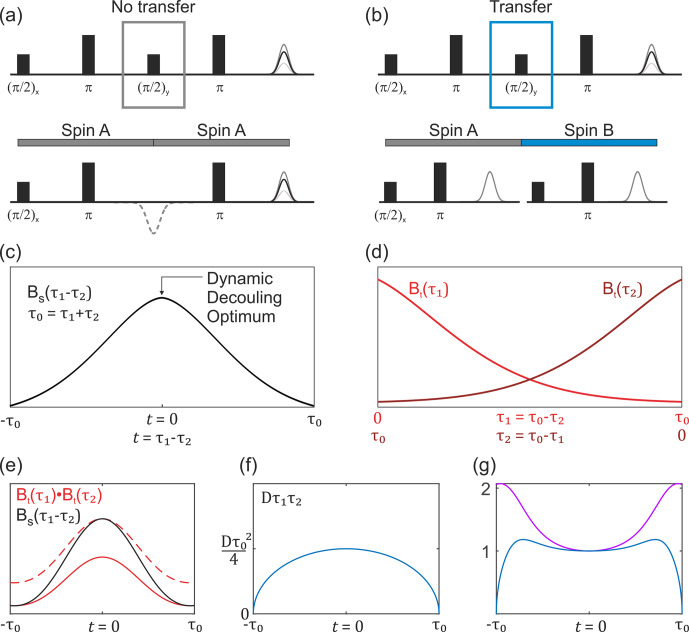
Overview of the buildup of the non-modulated part of the SIFTER signal. **(a)** SIDRE pulse sequence, which omits coherence transfer by the central $(\pi /2{)}_{y}$ pulse. **(b)** SIFTER pulse sequence with coherence transfer. The two respective pulse sequence blocks show a difference in evolution with and without coherence transfer by the central $(\pi /2{)}_{y}$ pulse. **(c)** Sketch of the SIDRE signal $B_{S}(\tau_{1},\tau_{2})$ with the point of optimal dynamic decoupling indicated by the arrow. **(d)** Sketch of two variable-time Hahn echo traces, which are combined into the second type of background term, valid in the case of coherence transfer as shown in **(b)**. **(e)** Comparison sketch of the SIDRE signal (black), combination of two variable-time Hahn echo decays (solid red line) and the double-Hahn echo decay signal rescaled to the same amplitude as the SIDRE signal at the time point t=0 (dashed red line). **(f)** Intermolecular coherence transfer factor $D\tau_{1}\tau_{2}$ as a function of the SIFTER time t . **(g)** Sketch of the divided trace $B_{t}(\tau_{1})B_{t}(\tau_{2})/B_{S}(\tau_{1},\tau_{2})$ (violet) and this signal multiplied by the intermolecular coherence transfer factor $D\tau_{1}\tau_{2}$ (blue). Note that $\tau_{0}=\tau_{1}+\tau_{2}$ , and for the SIFTER time $t=\tau_{1}-\tau_{2}$ .

For the intermolecular coherence transfer term (second term in Eq. [Disp-formula Ch1.E13]), the transverse relaxation will take place at two different spin centres during the two refocusing periods. Therefore, no pre-filtering can be assumed for the second transverse evolution period in the SIFTER sequence, unless the transverse evolution properties change very slowly over the spatial positions of electron spins and, therefore, correlate for the spins that are substantially coupled via dipolar interaction. Under the assumption of no such correlation, the other part of the transverse relaxation will be a product of two variable-time Hahn echo decays, similar to the intermolecular electron–electron dipolar terms in the above SIFTER calculations. We shall abbreviate this term as $B_{t}(\tau_{1})B_{t}(\tau_{2})$ ; see Fig. [Fig Ch1.F3]b and d. We shall use an abbreviation $B_{t}(\tau )$ to distinguish the Hahn echo decay that includes electron–electron and electron–nuclear contributions, as well as the distribution of intrinsic transverse relaxation times, from the pure electron dipolar contribution $B_{2p}(\tau )$ , which should not be prone to such filtering effects, assuming homogeneous solution. Note further that here we discuss the case where the main contribution to $B_{t}(\tau )$ is due to the electron–nuclear interactions, which introduce the filtering effects discussed above.

In the situation of filtering, the two terms $B_{S}(\tau_{1},\tau_{2})$ and $B_{t}(\tau_{1})B_{t}(\tau_{2})$ have different shapes. In a graphical representation, when these two traces are scaled to the same value at the point $\tau_{1}=\tau_{2}$ , somewhat counterintuitively, the second term would be decaying slower towards the outer borders of the $\tau_{1}-\tau_{2}$ region, as compared to the first term (Fig. [Fig Ch1.F3]e). This follows from the fact that at $\tau_{1}=0$ or $\tau_{2}=0$ the two terms are equal, while at the time point $\tau_{1}=\tau_{2}$ the unscaled term $B_{S}(\tau_{1},\tau_{2})$ assumes a larger value than the unscaled term $B_{t}(\tau_{1})B_{t}(\tau_{2})$ , as is also known from dynamical decoupling [Bibr bib1.bibx45]. In the case when the SIFTER experiment is performed on a frozen solution of monoradicals, there is no intramolecular dipolar term, and the overall SIFTER signal $V_{\mathrm{SIFTER}}^{\mathrm{mono}}(\tau_{1},\tau_{2})$ will consist of the contribution with no intermolecular coherence transfer, which will have the transverse evolution term of the form $B_{S}(\tau_{1},\tau_{2})$ , and the contribution with intermolecular coherence transfer, which will have the transverse evolution of the form $B_{t}(\tau_{1})B_{t}(\tau_{2})$ ; thus,

17 $$V_{\mathrm{SIFTER}}^{\mathrm{mono}}(\tau_{1},\tau_{2})=B_{S}(\tau_{1},\tau_{2})+D\tau_{1}\tau_{2}\cdot B_{t}(\tau_{1})B_{t}(\tau_{2})\;.$$

Here, we used the following additional abbreviation for the ensemble-averaged square of the intermolecular dipolar frequency: $D=\langle \omega_{n}^{2}\rangle$ . If we divide the SIFTER signal of the monoradical sample by the $B_{S}(\tau_{1},\tau_{2})$ signal, which can be measured independently, then the remaining signal will have the following form:

18 $$S^{\mathrm{mono}}(\tau_{1},\tau_{2})=1+D\tau_{1}\tau_{2}\cdot \frac{B_{t}(\tau_{1})B_{t}(\tau_{2})}{B_{S}(\tau_{1},\tau_{2})}.$$

For a short overall length of the SIFTER trace, the filtration effects should be weak, and the two signals $B_{S}(\tau_{1},\tau_{2})$ and $B_{t}(\tau_{1})B_{t}(\tau_{2})$ would have similar shapes. The coherence transfer factor

19 $$D\tau_{1}\tau_{2}=\frac{D}{4}\left[(\tau_{1}+\tau_{2}{)}^{2}-(\tau_{1}-\tau_{2}{)}^{2}\right]=\frac{D}{4}\left[\tau_{0}^{2}-t^{2}\right]$$

has a parabolic shape curved down, and it is equal zero at the points $\tau_{1}=0$ and $\tau_{2}=0$ , i.e. at $t=\pm \tau_{0}$ (see Fig. [Fig Ch1.F3]f). For a short overall length of the SIFTER trace, this coherence transfer factor might dominate in the overall shape of $S^{\mathrm{mono}}(\tau_{1},\tau_{2})$ , and then this trace would be curved down. At a certain length of the SIFTER trace, the difference in shape between $B_{S}(\tau_{1},\tau_{2})$ and $B_{t}(\tau_{1})B_{t}(\tau_{2})$ decay curves would become significant. Note that because of filtration and dynamical decoupling effects for the $B_{S}(\tau_{1},\tau_{2})$ curve, the ratio $B_{t}(\tau_{1})B_{t}(\tau_{2})/B_{S}(\tau_{1},\tau_{2})$ would increase towards the ends of the SIFTER trace. In the outermost regions of the SIFTER trace on its left and right borders, where either $\tau_{1}$ or $\tau_{2}$ is close to zero, this ratio will level up to some nearly constant value and, again, the overall down-curved shape might appear (Fig. [Fig Ch1.F3]g), which, however, experimentally would be in most cases masked by the strong increase of the noise in the divided trace $S^{\mathrm{mono}}(\tau_{1},\tau_{2})$ in these regions.

### 2.3 The SIFTER signal of a biradical

In this section, we will analyse the frozen biradical solution, for which the spin Hamiltonian that includes electron–electron and electron–nuclear parts can be written in the form

20 $$\begin{array}{rl} \hat{H} & =\omega_{0}{\hat{S}}_{z}{\hat{I}}_{z}^{\left(0\right)}+\sum_{l=1}^{N}\omega_{l}{\hat{S}}_{z}{\hat{I}}_{z}^{\left(l\right)}+\sum_{i=1}^{N/2}{\tilde{\omega }}_{2i-1}{\hat{I}}_{z}^{\left(2i-1\right)}{\hat{I}}_{z}^{\left(2i\right)}\\ & +\frac{1}{2}\sum_{i,j}{\tilde{\omega }}_{i,j}{\hat{I}}_{z}^{\left(i\right)}{\hat{I}}_{z}^{\left(j\right)}+{\hat{H}}_{e\text{-}n,n\text{-}n}. \end{array}$$

In the term $\sum_{i,j}{\tilde{\omega }}_{i,j}{\hat{I}}_{z}^{\left(i\right)}{\hat{I}}_{z}^{\left(j\right)}$ the summation on the indices i and j goes from 0 to N , excluding j=i and either j=i-1 or j=i+1 ; the latter would be the partner spin's index in the same biradical. For i=0 , only the index j=0 is excluded. Like for the monoradicals case, here we will also first consider just the case of electron–electron couplings (next two subsections) and then discuss the filtration effects due to the electron spin interactions with the nuclear bath. In the latter case, the same assumption of the dominating nuclear bath effect on the shape of the SIFTER intermolecular decay signal is implied.

#### 2.3.1 SIFTER in a frozen biradical solution: main term

In this and the next section we discuss in detail the evolution of the SIFTER signal in an ensemble of biradicals, i.e. in the case of a strong intramolecular coherence transfer. Let us define the density operator term $\hat{P}$ , which describes the result of intramolecular two-spin evolution in the $\tau_{1}$ - (π) - $\tau_{1}$ block:

21 $$\hat{P}=-{\hat{S}}_{y}\mathrm{cos}(\omega_{0}\tau_{1})+2{\hat{S}}_{x}{\hat{I}}_{z}^{\left(0\right)}\mathrm{sin}(\omega_{0}\tau_{1})\;.$$

In order to describe the density matrix evolution upon all dipolar couplings between A spin and B spins, we will also use the abbreviation

22 $$\hat{Q}=-{\hat{S}}_{x}\mathrm{cos}(\omega_{0}\tau_{1})+2{\hat{S}}_{y}{\hat{I}}_{z}^{\left(0\right)}\mathrm{sin}(\omega_{0}\tau_{1})\;.$$

After the $\tau_{1}$ - (π) - $\tau_{1}$ evolution block, three types of terms appear in the density matrix. The first term has the same operator form as for the isolated biradical with an additional factor:

23 $${\hat{\sigma }}_{1}=\hat{P}\prod_{l=1}^{N}\mathrm{cos}(\omega_{l}\tau_{1})\;.$$

The second type of terms appears if we let only one ${\hat{I}}_{z}^{\left(l\right)}$ operator mix in during the time evolution. Such terms will play an important role in the coherence transfer step. This part of the density matrix can be written as

24 $${\hat{\sigma }}_{2}=\sum_{n}\left(2\hat{Q}{\hat{I}}_{z}^{\left(n\right)}\mathrm{sin}(\omega_{n}\tau_{1})\prod_{l\neq n}\mathrm{cos}(\omega_{l}\tau_{1})\right).$$

The third type of operator terms summed up in ${\hat{\sigma }}_{3}$ contains all possible products which include two or more different ${\hat{I}}_{z}^{\left(l\right)}$ operators. We shall see that these terms do not contribute to the SIFTER echo signal.

Note again that the product $\prod_{l=1}^{N}\mathrm{cos}(\omega_{l}\tau_{1})$ describes the intermolecular dipolar contribution to the two-pulse echo formed at the time point $2\tau_{1}$ . The transverse relaxation and the nuclear spectral diffusion terms can be included into this term as additional factors, thus forming either the electron–electron dipolar contribution $B_{2p}(\tau_{1})$ or the overall two-pulse echo decay function $B_{t}(\tau_{1})$ . We shall stay for now with the electron–electron dipolar-only contribution $B_{2p}(\tau_{1})$ , and filtration effects for biradicals will be considered at the end of this derivation. In Eq. ([Disp-formula Ch1.E24]) we can add the missing factor $\mathrm{cos}(\omega_{n}\tau_{1})$ in the product and rewrite the equation to the form

25 $${\hat{\sigma }}_{2}=\sum_{n}\left(2\hat{Q}{\hat{I}}_{z}^{\left(n\right)}\mathrm{tan}(\omega_{n}\tau_{1})\prod_{l}\mathrm{cos}(\omega_{l}\tau_{1})\right).$$

Here, it is obvious that the product $\prod_{l}\mathrm{cos}(\omega_{l}\tau_{1})$ , again, can be substituted by the $B_{2p}(\tau_{1})$ function.

Next, we apply the $(\pi /2{)}_{y}$ pulse which causes the coherence transfer from A spin to B spins. Upon action of this pulse, the ${\hat{S}}_{y}$ operator stays unchanged (also in the operator products!), while the operator ${\hat{S}}_{x}$ transforms into $-{\hat{S}}_{z}$ . All ${\hat{I}}_{z}^{\left(l\right)}$ operators are transformed into ${\hat{I}}_{x}^{\left(l\right)}$ operators. All terms in ${\hat{\sigma }}_{3}$ are transformed into double- or multi-quantum coherences which cannot evolve into detectable terms in the last $\tau_{2}$ - (π) - $\tau_{2}$ evolution block. Part of ${\hat{\sigma }}_{2}$ transforms into anti-phase coherences of a form $2{\hat{S}}_{z}{\hat{I}}_{x}^{\left(l\right)}$ , which evolve into detectable terms over the $\tau_{2}$ - (π) - $\tau_{2}$ block. We will discuss these terms in the next subsection. The strongest contribution appears from the term ${\hat{\sigma }}_{1}$ (Eq. [Disp-formula Ch1.E23]), which can be written after ensemble averaging as

26 $$\langle {\hat{\sigma }}_{1}\rangle =\langle {\hat{\sigma }}_{s}(2\tau_{1}-\delta t)\rangle \cdot B_{2p}(\tau_{1}),$$

The operator ${\hat{\sigma }}_{s}(2\tau_{1}-\delta t)$ is the full intramolecular term described in Eq. ([Disp-formula Ch1.E4]). After the $(\pi /2{)}_{y}$ pulse and the $\tau_{2}$ - (π) - $\tau_{2}$ evolution block, this operator evolves into the two-spin SIFTER signal Eq. ([Disp-formula Ch1.E6]), and an additional two-pulse echo decay factor appears in front of it due to another period of evolution under the intermolecular dipolar coupling terms. Thus, the full SIFTER signal, excluding the intermolecular coherence transfer terms, can be written as

27 $$V(\tau_{1},\tau_{2})=F(\tau_{2}-\tau_{1})\cdot B_{2p}(\tau_{1})\cdot B_{2p}(\tau_{2})\;.$$

Here, $F(\tau_{2}-\tau_{1})$ is the intramolecular form factor, obtained by averaging Eq. ([Disp-formula Ch1.E6]) over all spin–spin distances and dipolar angles and by dropping the non-detectable anti-phase coherence terms.

#### 2.3.2 SIFTER in a frozen biradical solution: additional terms

Additional terms appear in the above calculation (Eq. [Disp-formula Ch1.E25]) as a result of coherence transfer from A spins to the remote B spins. Let us give some comments on properties of the corresponding signal. The relevant terms just after the coherence transfer step sum up as follows:

28 $$\begin{array}{rl} \hat{A}(2\tau_{1}+\delta t) & =\sum_{n}(2{\hat{S}}_{z}{\hat{I}}_{x}^{\left(n\right)}\mathrm{cos}(\omega_{0}\tau_{1})\mathrm{sin}(\omega_{n}\tau_{1})\\ & \times \prod_{l\neq n}\mathrm{cos}(\omega_{l}\tau_{1}))\;. \end{array}$$

For a particular B spin with the index n , we get after the second evolution block a detectable contribution of the form

29 $$\begin{array}{rl} {\hat{A}}^{\left(n\right)}(2\tau_{1}+2\tau_{2}) & ={\hat{I}}_{y}^{\left(n\right)}\mathrm{cos}(\omega_{0}\tau_{1})\mathrm{tan}(\omega_{n}\tau_{1})\mathrm{tan}(\omega_{n}\tau_{2})\\ & \cdot \mathrm{cos}({\tilde{\omega }}_{n}\tau_{2})\prod_{l}\mathrm{cos}(\omega_{l}\tau_{1})\prod_{m}\mathrm{cos}({\tilde{\omega }}_{n,m}\tau_{2}). \end{array}$$

This can be ensemble averaged and projected onto the detection operator ${\hat{I}}_{y}^{\left(n\right)}$ , which results in a detected signal of the following form:

30 $$\begin{array}{rl} & \langle \mathrm{Tr}\left({\hat{A}}^{\left(n\right)}(2\tau_{1}+2\tau_{2})\cdot {\hat{I}}_{y}^{\left(n\right)}\right)\rangle \\ & \;=F(\tau_{1})F(\tau_{2})B_{2p}(\tau_{1})B_{2p}(\tau_{2})D\tau_{1}\tau_{2}. \end{array}$$

Note that here we approximated $\mathrm{tan}(\omega_{n}\tau )$ as $\omega_{n}\tau_{1}$ , because intermolecular couplings are assumed to be weak. The transformation from Eq. ([Disp-formula Ch1.E29]) to Eq. ([Disp-formula Ch1.E30]) contains a step, where, as for monoradicals, we assume that ensemble averaging of the two products of cosine functions is uncorrelated:

31 $$\begin{array}{rl} & \langle \prod_{l}\mathrm{cos}(\omega_{l}\tau_{1})\prod_{m}\mathrm{cos}({\tilde{\omega }}_{n,m}\tau_{2})\rangle \\ & \;=\langle \prod_{l}\mathrm{cos}(\omega_{l}\tau_{1})\rangle \langle \prod_{m}\mathrm{cos}({\tilde{\omega }}_{n,m}\tau_{2})\rangle . \end{array}$$

The two products in this equation correspond to the initial molecule's surrounding, for which the intermolecular dipolar frequencies are marked as $\omega_{l}$ , and the surrounding of the molecule containing the spin n , for which the intermolecular dipolar frequencies are marked as ${\tilde{\omega }}_{n,m}$ . These two molecules, obviously, must be separated by a distance that is sufficiently short to allow for some substantial coherence transfer driven by the corresponding intermolecular dipolar coupling. Thus, many spins, which strongly contribute to the intermolecular background decay for one of the spins, will be strongly affecting the intermolecular background decay of the other spin as well. Strictly speaking, the absence of the correlation in Eq. ([Disp-formula Ch1.E31]) is only a phenomenological assumption, which would need to be proven, for example, by Monte Carlo simulations. Under this assumption of uncorrelated relaxation, it is possible to approximate the factorization rule for the SIFTER experiment in the form

32 $$V_{\mathrm{SIFTER}}=\left(F(\tau_{2}-\tau_{1})+A(\tau_{1},\tau_{2})\right)\cdot B_{\mathrm{SIFTER}}(\tau_{1},\tau_{2})\;,$$

with the intermolecular coherence transfer artefact $A(\tau_{1},\tau_{2})$ and intermolecular background $B_{\mathrm{SIFTER}}(\tau_{1},\tau_{2})$ contributions

33 $$A(\tau_{1},\tau_{2})=F(\tau_{1})F(\tau_{2})D\tau_{1}\tau_{2},$$

34 $$B_{\mathrm{SIFTER}}(\tau_{1},\tau_{2})=B_{2p}(\tau_{1})B_{2p}(\tau_{2}).$$

The term $A(\tau_{1},\tau_{2})$ is proportional to D which should scale proportional to the square of the spin concentration. This can be used as one of the possible experimental checks for the validity of the presented theoretical description. However, this might appear complicated in practice because of the anticipated fast overall SIFTER signal decay at high spin concentrations.

Finally, we need to demonstrate that all density operator terms, which include more than one ${\hat{I}}_{z}$ operator at the point just before the central $(\pi /2{)}_{y}$ pulse, would not lead to any detectable terms in the SIFTER signal. After the $(\pi /2{)}_{y}$ pulse, such terms, which also include the ${\hat{S}}_{x}$ operator, will be transformed into a product of ${\hat{S}}_{z}$ operator with two or more ${\hat{I}}_{x}$ operators. During the following $\tau_{2}$ - $(\pi {)}_{x}$ - $\tau_{2}$ block, the evolution upon the action of one $2{\hat{S}}_{z}{\hat{I}}_{z}$ operator would remove the ${\hat{S}}_{z}$ from the product, and leave a product of at least two ${\hat{I}}_{k}^{\left(i\right)}$ operators with different i and k=x,y . Such terms will commute with the secular parts of the remaining dipolar interactions, and, thus, will not further evolve into detectable single-quantum coherences. The terms, which include the ${\hat{S}}_{y}$ operator, after the $(\pi /2{)}_{y}$ pulse, will immediately result in the multiple-quantum coherence terms that would not evolve under the secular parts of the dipolar couplings.

#### 2.3.3 Transverse evolution filtering in the biradical case

Here, as in the monoradical case, we will have to consider pathways with and without coherence transfer, as depicted in Fig. [Fig Ch1.F3]a–d. The analysis of the filtration effects for the frozen solution of biradicals follows the same general lines as in the monoradical case but with some important differences in the final equations. Again, for the main term that includes the intramolecular dipolar signal, the $\mathrm{cos}(\omega_{\mathrm{dd}}\tau_{1})\mathrm{cos}(\omega_{\mathrm{dd}}\tau_{2})$ term (see Eq. [Disp-formula Ch1.E7]) will have the transverse relaxation contribution $B_{S}(\tau_{1},\tau_{2})$ . For the $\mathrm{sin}(\omega_{\mathrm{dd}}\tau_{1})\mathrm{sin}(\omega_{\mathrm{dd}}\tau_{2})$ term of the intramolecular dipolar contribution (Eq. [Disp-formula Ch1.E8]) as well as for the intermolecular coherence transfer term, the transverse relaxation will take place at two different spin centres during the two refocusing periods. Therefore, no pre-filtering can be assumed for the second transverse evolution period in the SIFTER sequence, unless the transverse evolution properties change very slowly over the spatial positions of electron spins, and therefore correlate for the spins that are substantially coupled via dipolar interaction. Under the assumption of no such correlation, the other part of the transverse relaxation will be a product of two variable-time Hahn echo decays $B_{t}(\tau_{1})B_{t}(\tau_{2})$ .

In the situation of filtering, the two terms $B_{S}(\tau_{1},\tau_{2})$ and $B_{t}(\tau_{1})B_{t}(\tau_{2})$ have different shapes, with the second term decaying slower towards the outer borders of the $\tau_{1}-\tau_{2}$ region, as compared to the first term. This leads to a modification of the intramolecular as well as intermolecular SIFTER contributions and of the way these two contributions can be factorized. The $\mathrm{cos}(\omega_{\mathrm{dd}}(\tau_{1}-\tau_{2}))$ term we have encountered in Eq. ([Disp-formula Ch1.E6]) will be multiplied with the $B_{S}(\tau_{1},\tau_{2})$ term, while there will appear another term, containing only the product of two sine functions, multiplied by the difference of the two transverse relaxation terms: $\mathrm{sin}(\omega_{\mathrm{dd}}\tau_{1})\mathrm{sin}(\omega_{\mathrm{dd}}\tau_{2})\cdot \left[B_{t}(\tau_{1})B_{t}(\tau_{2})-B_{S}(\tau_{1},\tau_{2})\right]$ . The overall SIFTER signal for biradicals will be thus described by the following equation:

35 $$\begin{array}{rl} V_{\mathrm{SIFTER}} & =F(\tau_{2}-\tau_{1})\cdot B_{S}(\tau_{1},\tau_{2})\\ & +F_{s}(\tau_{1},\tau_{2})\cdot \left[B_{t}(\tau_{1})B_{t}(\tau_{2})-B_{S}(\tau_{1},\tau_{2})\right]\\ & +A(\tau_{1},\tau_{2})\cdot B_{t}(\tau_{1})B_{t}(\tau_{2})\;, \end{array}$$

and, after dividing $V_{\mathrm{SIFTER}}$ by the SIDRE signal $B_{S}(\tau_{1},\tau_{2})$ ,

36 $$\begin{array}{rl} S_{\mathrm{SIFTER}} & =F(\tau_{2}-\tau_{1})\\ & +F_{s}(\tau_{1},\tau_{2})\cdot \frac{B_{t}(\tau_{1})B_{t}(\tau_{2})-B_{S}(\tau_{1},\tau_{2})}{B_{S}(\tau_{1},\tau_{2})}\\ & +A(\tau_{1},\tau_{2})\cdot \frac{B_{t}(\tau_{1})B_{t}(\tau_{2})}{B_{S}(\tau_{1},\tau_{2})}. \end{array}$$

Here, we used an abbreviation $F_{s}(\tau_{1},\tau_{2})$ for the artefact signal composed of sine contributions:

37 $$F_{s}(\tau_{1},\tau_{2})=\langle \mathrm{sin}(\omega_{\mathrm{dd}}\tau_{1})\mathrm{sin}(\omega_{\mathrm{dd}}\tau_{2})\rangle .$$

Using the trigonometric relation $\mathrm{sin}\alpha \mathrm{sin}\beta =\frac{1}{2}\left[\mathrm{cos}(\alpha -\beta )-\mathrm{cos}(\alpha +\beta )\right]$ , this can be transformed to

38 $$\begin{array}{rl} F_{s}(\tau_{1},\tau_{2}) & =\frac{1}{2}\langle \mathrm{cos}[\omega_{\mathrm{dd}}(\tau_{1}-\tau_{2})]\rangle -\langle \mathrm{cos}[\omega_{\mathrm{dd}}(\tau_{1}+\tau_{2})]\rangle \\ & =\frac{1}{2}F(\tau_{2}-\tau_{1})-\frac{1}{2}\langle \mathrm{cos}[\omega_{\mathrm{dd}}(\tau_{1}+\tau_{2})]\rangle . \end{array}$$

Note that here the first term is equal to the normal SIFTER intramolecular signal, and the second term is constant at any time point for a given total length of the SIFTER trace. Equation ([Disp-formula Ch1.E36]) can thus be rewritten in the form

39 $$\begin{array}{rl} S_{\mathrm{SIFTER}} & =F(\tau_{2}-\tau_{1})\cdot \left[1+\frac{B_{t}(\tau_{1})B_{t}(\tau_{2})-B_{S}(\tau_{1},\tau_{2})}{2B_{S}(\tau_{1},\tau_{2})}\right]\\ & +\langle \mathrm{cos}[\omega_{\mathrm{dd}}(\tau_{1}+\tau_{2})]\rangle \cdot \frac{B_{t}(\tau_{1})B_{t}(\tau_{2})-B_{S}(\tau_{1},\tau_{2})}{2B_{S}(\tau_{1},\tau_{2})}\\ & +A(\tau_{1},\tau_{2})\cdot \frac{B_{t}(\tau_{1})B_{t}(\tau_{2})}{B_{S}(\tau_{1},\tau_{2})}\;. \end{array}$$

The first term in this equation can be further rewritten as

40 $$F(\tau_{2}-\tau_{1})\cdot \frac{1}{2}\left[1+\frac{B_{t}(\tau_{1})B_{t}(\tau_{2})}{B_{S}(\tau_{1},\tau_{2})}\right]\;,$$

which corresponds to the main dipolar evolution term in the SIFTER signal before division by $B_{S}(\tau_{1},\tau_{2})$ in the form

41 $$V_{\mathrm{SIFTER}}^{\mathrm{main}}=F(\tau_{2}-\tau_{1})\cdot \frac{1}{2}\left[B_{S}(\tau_{1},\tau_{2})+B_{t}(\tau_{1})B_{t}(\tau_{2})\right].$$

The function in the square brackets describes the intermolecular contribution factor for the intramolecular dipolar modulation in the SIFTER trace.

To summarize, the detected SIFTER signal contains a modulated and a non-modulated part:

42 $$V_{\mathrm{SIFTER}}=(1-\lambda )\cdot V_{\mathrm{SIFTER}}^{n.m.}+\lambda \cdot V_{\mathrm{SIFTER}}^{\mathrm{mod}.}.$$

The non-modulated part of the SIFTER signal of biradicals exhibits the form

43 $$\begin{array}{rl} V_{\mathrm{SIFTER}}^{n.m.}(\tau_{1},\tau_{2}) & =B_{S}(\tau_{1},\tau_{2})\\ & \cdot \left(1-\frac{\lambda }{2(1-\lambda )}\langle \mathrm{cos}[\omega_{\mathrm{dd}}(\tau_{1}+\tau_{2})]\rangle \right)\\ & +B_{t}(\tau_{1})B_{t}(\tau_{2})\\ & \cdot \left(D\tau_{1}\tau_{2}+\frac{\lambda }{2(1-\lambda )}\langle \mathrm{cos}[\omega_{\mathrm{dd}}(\tau_{1}+\tau_{2})]\rangle \right)\;, \end{array}$$

with λ being the modulation depth in the SIFTER trace. Note the very similar shape of this signal to the SIFTER signal in the case of monoradicals. Accordingly, it will have the same properties depicted in the Fig. [Fig Ch1.F3]e–g. The dipolar modulated part of the SIFTER signal (including artefact and omitting λ scaling) has the form

44 $$\begin{array}{rl} V_{\mathrm{SIFTER}}^{\mathrm{mod}.} & =F(\tau_{2}-\tau_{1})\cdot \frac{1}{2}\left[B_{S}(\tau_{1},\tau_{2})+B_{t}(\tau_{1})B_{t}(\tau_{2})\right]\\ & +F(\tau_{1})F(\tau_{2})D\tau_{1}\tau_{2}\cdot \left[B_{t}(\tau_{1})B_{t}(\tau_{2})\right]\;. \end{array}$$

Here, again, the intermolecular background contributions are written in square brackets. The artefact (second term) might be of importance for the intramolecular dipolar signals with long-lasting dipolar oscillations. In the more common cases of quickly decaying intramolecular dipolar signal, this artefact should be weak at nearly all times.

## 3 Materials and methods

Nitroxide biradical (3) [Bibr bib1.bibx39], trityl biradical (4) [Bibr bib1.bibx50] and trityl monoradical (2) [Bibr bib1.bibx17] were synthesized as described in the given references. 2,2,6,6-Tetramethylpiperidinyloxyl (TEMPO) (1) of analytical purity was obtained from Sigma-Aldrich (Buchs, Switzerland). All compounds were dissolved in *ortho*-terphenyl (OTP, Sigma-Aldrich), transferred into 1.6 mm outer diameter quartz capillaries (Wilmad-LabGlas), melted at 80 °C before flash freezing in liquid nitrogen to ensure homogeneous glass formation. All samples were measured at a spin concentration of 50 µ M, except (1) which was measured at 100 µ M.

EPR measurements were performed on a home-built high-power (150 W traveling-wave tube amplifier) Q-band spectrometer [Bibr bib1.bibx11] in a fully overcoupled home-built pent-loop gap resonator [Bibr bib1.bibx48] at a temperature of 80 K. Where not otherwise stated, measurements were performed with both π/2 and π Gaussian pulses of 64 ns. For nitroxides, additional experiments were performed using rectangular and frequency-swept pulses. Rectangular π/2 and π pulses were both 6 ns in length with no further compensations. Hyperbolic secant pulses (asymmetric order 1 and 6) were 128 ns long and were compensated for the experimental resonator profile [Bibr bib1.bibx12]. Excitation was centred at the spectral maximum in all experiments. Shot repetition times under all conditions were chosen to provide >98 % signal recovery in inversion recovery experiments to avoid significant $T_{1}$ contributions to relaxation behaviour. Field sweeps, two-pulse (Hahn) decays and inversion recovery were measured with a standard two-step phase cycle. SIFTER and derived experiments were recorded with a 16-step phase cycle [Bibr bib1.bibx24]. All experimental data were recorded in transient form, and echoes were integrated over a 128 ns window. Data of two-pulse decays were fitted by a stretched exponential function of the form $A=\mathrm{exp}(-(\frac{t}{\tau }{)}^{\beta })$ or by a sum of stretched exponentials (SSEs).

## 4 Discussion and possibilities of validation

In this work we derived analytic equations for the SIFTER signal in frozen glassy solutions of monoradicals and biradicals. Importantly, in this analysis we obtained the SIFTER signal for monoradicals as a sum of two well defined contributions that can be also independently determined in auxiliary measurements. Also for biradicals, we determined the dipolar modulated part of the SIFTER signal to consist of two terms, each presented as a product of an intramolecular contribution and an intermolecular contribution. Moreover, the analysis suggests that the main signal, which represents the classical intramolecular dipolar evolution signal, has a well defined intermolecular contribution that can be determined by the SIDRE experiment and variable delay Hahn echo experiments. This signal (first term in Eq. [Disp-formula Ch1.E44]) is also expected to have significantly stronger intensity than the other contribution. Indeed, the intermolecular dipolar evolution artefacts at the two ends of the SIFTER trace (second term in Eq. [Disp-formula Ch1.E44]) would be suppressed by the weakness of the intermolecular dipolar coupling (provided that, as usual in pulse EPR, samples with low spin concentrations are used), and, additionally, by the inverted parabolic factor $\tau_{1}\tau_{2}$ which is equal to zero on both ends of the SIFTER time trace, i.e. exactly at the points where the corresponding dipolar evolution factors F(τ) should otherwise have the highest amplitude. Importantly, this artefact contribution should increase proportionally to the square of the spin concentration.

Note also that the relative contributions of the artefact term should not depend on the thermal Boltzmann polarization of the spins, since this only affects the initial polarization of the spin system, but it does not influence any steps in the presented density matrix propagation. Thus, intensities of all terms in the final equations would simply scale linearly with the Boltzmann polarization, and their ratios would remain unaffected.

There is, also, another important effect that makes the amplitude of the artefact signal $F(\tau_{1})F(\tau_{2})$ significantly smaller, as compared to the main SIFTER signal $F(\tau_{1}-\tau_{2})$ . Since the artefact term is a product of two dipolar evolution signals, each dependent only on one delay time $\tau_{1}$ or $\tau_{2}$ , if we formally fix one delay time and vary the other one, the maximum intensity of the signal will be at the point τ=0 . The signal will decay towards the end of the trace and reach its minimum value when τ reaches its maximum value. Now, if we assume the correlated change of the two delay times, as in the SIFTER experiment, we realize that the maximum of one part of the product will correspond to the minimal amplitude of the other part of the product: $\tau_{1}=max$ corresponds to $\tau_{2}=0$ and vice versa. Therefore, the artefact $F(\tau_{1})F(\tau_{2})$ can become significant only if the characteristic decay time of the dipolar evolution trace is comparable with the full length of the SIFTER trace. Obviously, regardless of the presence or absence of the artefact, this length of the SIFTER trace will also mean that such a trace would be too short to compute accurately the corresponding distance distribution. Thus, we can conclude that in most of the practically useful SIFTER measurements the presence of the second term in the SIFTER signal, described by Eq. ([Disp-formula Ch1.E44]), would introduce only very weak trace distortions, which should not significantly affect accuracy of the SIFTER data analysis.

The quantitative analysis of the structure of the intramolecular SIFTER signal, and validation of the presented analytical solution requires substantial effort and needs good quality reference data on the “true distance distribution” in the sample under study (e.g. measured by DEER). Here, we will concentrate on the analysis of the intermolecular SIFTER signal in monoradical solutions and the non-modulated part of the SIFTER signal of biradicals. These contributions should be described by Eqs. ([Disp-formula Ch1.E17]) and ([Disp-formula Ch1.E18]) and by Eq. ([Disp-formula Ch1.E43]), respectively. Note that for proper distance distribution analysis, according to our equations, the removal of the non-modulated contribution in SIFTER should be performed by fitting and subtraction rather than by division as in DEER. Of course, after such subtraction, the modulated part of the SIFTER signal would still need to be divided by the appropriate (different in shape) biradical-related background function $\frac{1}{2}\left[B_{S}(\tau_{1},\tau_{2})+B_{t}(\tau_{1})B_{t}(\tau_{2})\right]$ (as described in Eq. [Disp-formula Ch1.E44] for the main, first term). We would like to reiterate here that the currently used heuristic background correction procedure, based on the analogy with DEER data analysis, unfortunately, does not match our theoretical predictions. The intermolecular background signal for the modulated part of SIFTER signal would decay slower than the SIDRE signal $B_{S}(\tau_{1},\tau_{2})$ . Therefore, background division with using SIDRE as a background function or with fitting the background function to the unmodulated part of the SIFTER signal would disturb the desired intramolecular dipolar modulations.

For our current purpose of validation of theory, however, it is more convenient to divide both monoradical and biradical SIFTER data by the corresponding SIDRE traces and compare the obtained shapes with the shapes of the division traces

$$B_{t}(\tau_{1})B_{t}(\tau_{2})/B_{S}(\tau_{1},\tau_{2}).$$

The similarity in the shapes in two such series would, first, confirm the above assumption of the uncorrelated intermolecular contributions from the dipolar coupled spins in the coherence transfer terms in the SIFTER signal. Second, in the case of biradical SIFTER traces, such a comparison would also confirm our result related to the composition of the SIFTER signal as a sum of “monoradical-like” and “biradical-like” contributions.

## 5 Experimental results and discussion

Experimental SIFTER traces exhibit a characteristic dependence of their background shape on the trace length (see Fig. [Fig Ch1.F4]a and d), where shorter traces have a uniformly curved shape and, with increasing trace length, the shape gradually shifts to a more Gaussian form. However, while qualitatively similar, this is characteristically different between trityl and nitroxide. The overall decay rate, characterized by the relative loss of signal when stepping out of the zero-time condition, increases with trace length for nitroxide, whereas it decreases for trityl. It should be noted that the effect is generally much more prominently visible for nitroxide than trityl. Analogous trends can be seen for the SIDRE experiment (panels b and e). Division of the SIFTER traces by the corresponding SIDRE traces (panels c and f), as has been suggested earlier to be performed for partial background correction [Bibr bib1.bibx6], does result in significantly flatter shapes with relaxation contributions removed. Again the observed shape depends on trace length, here flattening further as traces increase in length.

**Figure 4 Ch1.F4:**
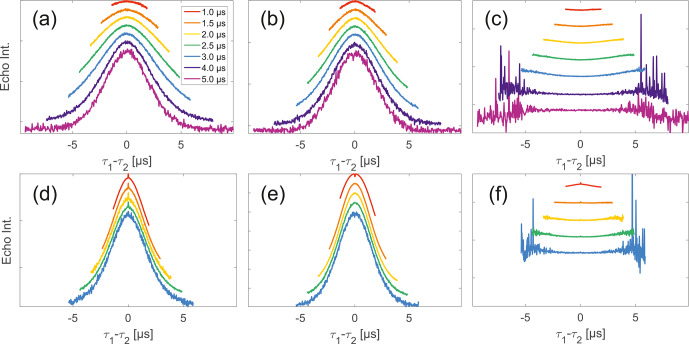
Analysis of data from SIFTER and SIDRE at various trace lengths on 50 µ M monoradicals in OTP. Panels **(a)**–**(c)** show data of nitroxide monoradical (1); **(d)**–**(f)** show data of trityl monoradical (2). The recorded traces of SIFTER **(a, d)** and SIDRE **(b, e)** are shown. The remaining panels **(c, f)** show the result from division of SIFTER by the corresponding SIDRE trace. Traces in **(c)** and **(f)** are displayed in stack plots at arbitrary offset.

Observing a two-pulse echo in comparison to the refocused echo, we predominantly find an upward curving of the divided traces (Fig. [Fig Ch1.F5]) similar to our comparison of division of SIFTER by SIDRE (Fig. [Fig Ch1.F4]). Again the only exception observed is the shortest trace recorded on trityl monoradical in which case a slight downward curvature is observed (and can be speculatively attributed to the domination of the $D\tau_{1}\tau_{2}$ factor in the intermolecular coherence transfer term). The SIDRE-divided longer traces become significantly flatter for both compounds, suggesting the characteristic difference in the shape of SIDRE, representing $B_{S}$ , and the two-pulse decay product, representing $B_{\mathrm{SIFTER}}$ , is reduced here. The similarity of the traces resulting from division in Fig. [Fig Ch1.F4]c and f compared to Fig. [Fig Ch1.F5]c and f is entirely consistent with the prediction made in Eqs. ([Disp-formula Ch1.E17]) and ([Disp-formula Ch1.E18]). First, we observe in Fig. [Fig Ch1.F4] that all traces are flatter after division by the corresponding SIDRE traces. While this is consistent with the idea of removal of part of the background, it is in itself not a convincing argument for validity of the theory as the same would be true for division by any decaying signal. Rather, the information supporting our theory lies within the dependence on trace length and in conformity with our expectation of the relative behaviour of the three contributions $B_{S}(\tau_{1},\tau_{2})$ , $B_{t}(\tau_{1})B_{t}(\tau_{2})$ and $D\tau_{1}\tau_{2}$ . Between the first two contributions we expect different relaxation behaviour based on dynamical decoupling arguments. The more efficient coherence recovery in $B_{S}(\tau_{1},\tau_{2})$ at $\tau_{1}\approx \tau_{2}$ , i.e. near the centre of the trace, results in faster decay when increasing the difference between $\tau_{1}$ and $\tau_{2}$ compared to $B_{t}(\tau_{1})B_{t}(\tau_{2})$ , where no such dynamical decoupling effect is contained. Therefore, an upward curvature of the divided traces is expected, as can be seen in Fig. [Fig Ch1.F5]. The effect is visible in all traces in panel (c), i.e. nitroxide and all but the shortest for trityl. Towards the outer edges of the traces this difference between $B_{S}$ and $B_{t}$ will become minimal as $\tau_{1}$ and $\tau_{2}$ are so dissimilar that dynamical decoupling is no longer of relevance. This is expected to be more prominently visible in longer traces which we appropriately observe to flatten towards their outer edges. The effect can be verified in Fig. [Fig Ch1.F5]c and f. In the case of nitroxide (panel c) a general flattening is observed for the two longest traces, in the case of trityl (panel f) this general flattening is observed only for the longest trace but flattening off at the edges of the traces is observed also for shorter traces. The second trace length effect we expect is related to the artefact term $D\tau_{1}\tau_{2}$ . While the term itself according to Eq. ([Disp-formula Ch1.E18]) should always have a parabolic shape, its contribution is scaled by $B_{t}(\tau_{1})B_{t}(\tau_{2})$ . When the signal for either of the $B_{t}$ terms is decayed, the contribution of the coherence transfer artefact becomes negligible. Therefore, we expect it to contribute predominantly in short traces. The associated downward curvature along the trace can be seen for the shortest trace measured on trityl in Fig. [Fig Ch1.F7]f.

**Figure 5 Ch1.F5:**
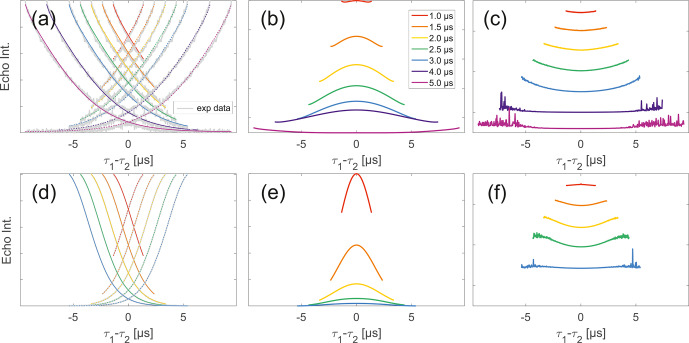
Analysis of two-pulse decay and refocussed echo data on 50 µ M monoradicals in OTP. Panels **(a)**–**(c)** show data of nitroxide monoradical (1); **(d)**–**(f)** show data of trityl monoradical (2). two-pulse decays with corresponding SSE fits (grey), mirrored and aligned to reflect offsets $\tau_{1}$ and $\tau_{2}$ in the SIFTER experiment **(a, d)**, product of the fits of the aligned decay traces **(b, e)** and result from division by corresponding SIDRE traces **(c, f)** (reflecting $B_{S}$ ) are shown. Traces in **(c)** and **(f)** are displayed in stack plots at arbitrary offset.

Stepping away from the monoradicals, we perform the same analysis for biradicals (Figs. [Fig Ch1.F6] and [Fig Ch1.F7]). Importantly, in agreement with the argumentation in the previous section, no significant dipolar evolution artefacts are visible at the outer parts of the SIFTER traces for the biradical samples. Based on our derivation we would expect trends described by Eqs. ([Disp-formula Ch1.E35]) and ([Disp-formula Ch1.E36]) for the observed background in biradicals, under the assumption of ideal pulses. Due to the selective pulse setup used here, we violate this assumption experimentally, which becomes apparent in observed low modulation depths of SIFTER traces and, accordingly, the unmodulated part of the background should be in line with the monoradical solution (Eqs. [Disp-formula Ch1.E17], [Disp-formula Ch1.E18]). For both nitroxide and trityl biradicals, we do observe that SIDRE data (Fig. [Fig Ch1.F6]b and e), which represent the $B_{S}$ term, appear to reflect the background decay observed in SIFTER data (Fig. [Fig Ch1.F6]a and d) rather well with the exception of short traces on trityl radicals.

**Figure 6 Ch1.F6:**
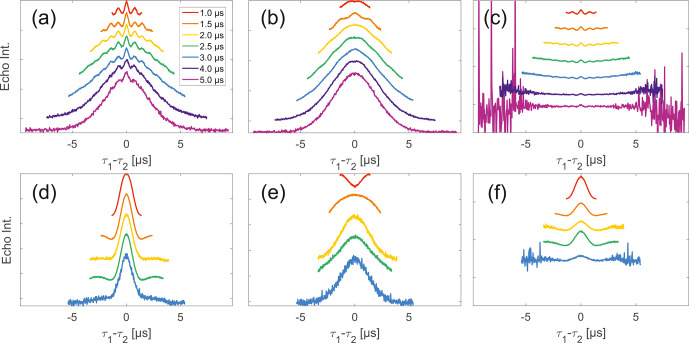
Analysis of data from SIFTER and SIDRE at various trace lengths on 50 µ M biradicals in OTP. Panels **(a)**–**(c)** show data of nitroxide biradical **3**; **(d)**–**(f)** show data of trityl biradical **4**. The recorded traces of SIFTER **(a, d)**, and SIDRE **(b, e)** are shown. The remaining panels **(c, f)** show the result from division of SIFTER by the corresponding SIDRE traces. Traces in **(c)** and **(f)** are displayed in stack plots at arbitrary offset.

**Figure 7 Ch1.F7:**
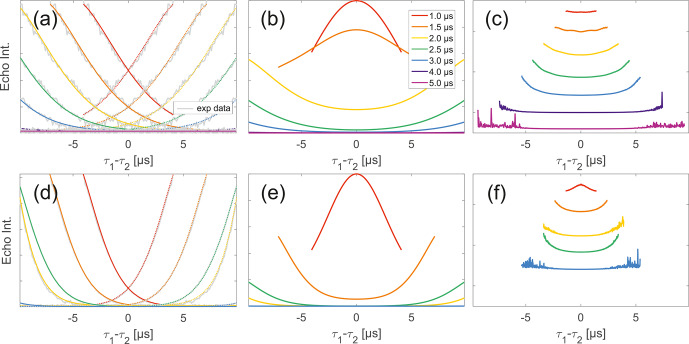
Analysis of two-pulse decay and refocussed echo data on 50 µ M biradicals in OTP. Panels **(a)**–**(c)** show data of nitroxide biradical (3); **(d)**–**(f)** show data of trityl biradical (4). Two-pulse decays with corresponding SSE fits (grey), mirrored and aligned to reflect offsets $\tau_{1}$ and $\tau_{2}$ in SIFTER experiment **(a, d)**, product of the fits of the aligned decay traces **(b, e)** and result from division by corresponding SIDRE trace **(c, f)** (reflecting $B_{S}$ ) are shown. Traces in **(c)** and **(f)** are displayed in stack plots at arbitrary offset.

While many of the trends just described for nitroxides remain identical to what we have described for monoradicals we observed prominent additional effects that are not covered by our model. We will attempt to ignore the prominent oscillations visible in the SIFTER traces, especially of nitroxide (Fig. [Fig Ch1.F6]), as they result from the primary dipolar signal and are thus not relevant to a discussion of the background. These oscillations also feature in Fig. [Fig Ch1.F6]c and f for the same reason. More interestingly, oscillations are visible in the SIDRE of nitroxide (Fig. [Fig Ch1.F6]b), which disappear with long trace lengths. These oscillations most probably result from other dipolar pathways due to imperfect pulses as detailed in earlier work [Bibr bib1.bibx11]. This is consistent with the dependence on trace length and the distortions of modulation intensity visible in the corresponding SIFTER traces. No such effect is observed in trityl biradical, where, however, the SIDRE features the largest observed shape changes of all samples studied (Fig. [Fig Ch1.F6]e), with substantially increased signal at the ends of the traces rather than the symmetrical, CPMG (Carr–Purcell–Meiboom–Gill) dynamical decoupling condition, where all other traces exhibit a maximum. We do currently not understand what causes this, but would like to point out that the flattening in division traces, as described for monoradicals, can still be observed in Fig. [Fig Ch1.F7]c and f as well as Fig. [Fig Ch1.F6]c and f. In the case of trityls, the selective pulse setup should still excite the majority of spins. As a result, we see significantly larger modulation depths than in the case of nitroxides. As a result of this, combined with the slow oscillations resulting from the long distance in the model compound, it becomes difficult to judge the background visually. While division by SIDRE (Fig. [Fig Ch1.F6]f) appears to flatten the traces, the shapes of the different traces obtained after this correction do not appear to show a systematic trend with increasing trace lengths, partially due to very strong and slow modulations. However, also for trityls we suggest that the observed background behaviour does not contradict the model of a combination of biradical and monoradical contributions.

## 6 Conclusions

Overall, there is a good match between the shapes of SIFTER data divided by the SIDRE traces ( $B_{S}(\tau_{1},\tau_{2})$ ) and the traces $B_{t}(\tau_{1})B_{t}(\tau_{2})/B_{S}(\tau_{1},\tau_{2})$ of the same length. This supports our assumption that the intermolecular dipolar evolution traces as well as the overall transverse evolution traces of different spins within the dipolar coupling range can be treated as uncorrelated. Additionally, the mentioned match of the shapes of divided traces indicates that the SIFTER signal measured on biradicals can be represented indeed as a sum of a biradical contribution, which is modulated with intramolecular dipolar oscillations, and a monoradical-like contribution, which has essentially the same structure and properties as the SIFTER signal of monoradicals. Our theoretical results predict that the modulated SIFTER signal is multiplied with one type of the intermolecular background function, while the unmodulated part of the SIFTER signal, which is also an intermolecular signal on its own, has a different shape. Accordingly, the appropriate background correction requires fitting and subtraction of the unmodulated part, followed by a division by a different background function, distinct from the unmodulated SIFTER signal.

Also, as predicted by the analytic equations, while some dipolar evolution artefacts must be present in SIFTER data, their relative contributions are very weak for most of the practically important cases. This prediction matches with the presented experimental SIFTER data, where such artefacts were not observed. Thus, the analytic approach proposed here, appears to be accurate to a good approximation. This opens up the possibility of a more detailed analysis of intramolecular SIFTER data, and quantitative evaluation and accuracy estimates of the distance distributions obtained from SIFTER measurements. Due to the complexity of the background problem outlined here, concomitant fitting of the modulated SIFTER signal and background will be an advantage, as recently shown for DEER [Bibr bib1.bibx18].

## Data Availability

Experimental data as well as scripts for data processing are made available via Zenodo with the following DOI: 10.5281/zenodo.7113575 (Vanas et al., 2022).
